# Urinary bladder diverticulum as a content of femoral hernia: a case report and review of literature

**DOI:** 10.1186/1749-7922-8-20

**Published:** 2013-06-11

**Authors:** Abdelkarim Hussein Omari, Mohammad Ahmad Alghazo

**Affiliations:** 1 Department of General Surgery, King Abdullah University Hospital, Faculty of Medicine, Jordan University of Science and Technology, Irbid, 22110, Jordan; 2 Division of urology, King Abdullah University Hospital, Faculty of Medicine, Jordan University of Science and Technology, Irbid, 22110, Jordan; 3 Department of General Surgery and Urology, Faculty of Medicine, Jordan University of Science and Technology, PO Box 3030, Irbid, 22110, Jordan

**Keywords:** Femoral hernia, Urinary bladder diverticula, Cystogram, Bladder outlet obstruction

## Abstract

**Background:**

Long standing increase of the intravesical pressure resulting from urinary bladder outlet obstruction can cause both secondary bladder diverticula and groin hernias. In rare cases, a diverticulum can be pulled by a hernia sac and becomes a component of the hernia itself. Such cases were encountered in inguinal, perineal and obturator hernias. However, to our knowledge, there has been only one case reported in the literature of a bladder diverticulum herniated in to the femoral canal.

**Methods:**

Literature search using PubMed was performed to identify all published cases of herniation of bladder diverticula in to the femoral canal.

**Results:**

Literature search revealed only one case before the present one.

**Conclusion:**

Urinary bladder diverticula should be considered as a possible content of femoral hernias especially in males with long standing obstructive lower urinary tract symptoms. As the clinical features of such a case are not specific, a high index of suspicion along with proper imaging studies are of great help in making a timely diagnosis to improve the outcome.

## Introduction

Groin hernia is a common surgical disease and its content is usually intra-abdominal viscera surrounded by the peritoneum. An extra peritoneal organ cannot be contained in the sac of the hernia. However, it can be pulled by the sac itself and becomes a component of the hernia as in the case of a bladder diverticulum [1].

Femoral hernias are less common than inguinal hernias and are usually complicated with incarceration or strangulation of the organ that they contain [2,3]. Bladder diverticula arise within a trabeculated high pressure urinary bladder caused by bladder outlet obstruction. In most cases, it is a result of benign prostatic hypertrophy. As the clinical features of the bladder diverticulum are not specific, high index of suspicion along with proper imaging studies are of great help in making a timely diagnosis.

We present a case of a huge urinary bladder diverticulum that herniated into the right femoral canal in association with indirect reducible right inguinal hernia.

### Case report

A 59-year old obese man presented to the emergency department with a long standing history of lower urinary tract symptoms and a subsequent appearance of a right groin swelling of nine months duration. His symptoms of difficulty of urination, increased urinary frequency, nocturia and urgency became worse when the groin swelling increased in size. The patient used to reduce the swelling manually to improve the symptoms. Six hours prior to the emergency room visit, the pain became intolerable and the swelling was tender and irreducible. The patient has essential hypertension and benign prostatic hypertrophy for the last 5 years.

Physical examination revealed that the patient had stable vital signs and controlled blood pressure. Body mass index (BMI) was 32 kg/m2. Abdominal examination showed the presence of a tender right groin swelling which was difficult to assess because of tenderness and obesity. Digital rectal examination showed a clinically benign enlarged prostate about 80 grams in volume.

**Figure 1:**
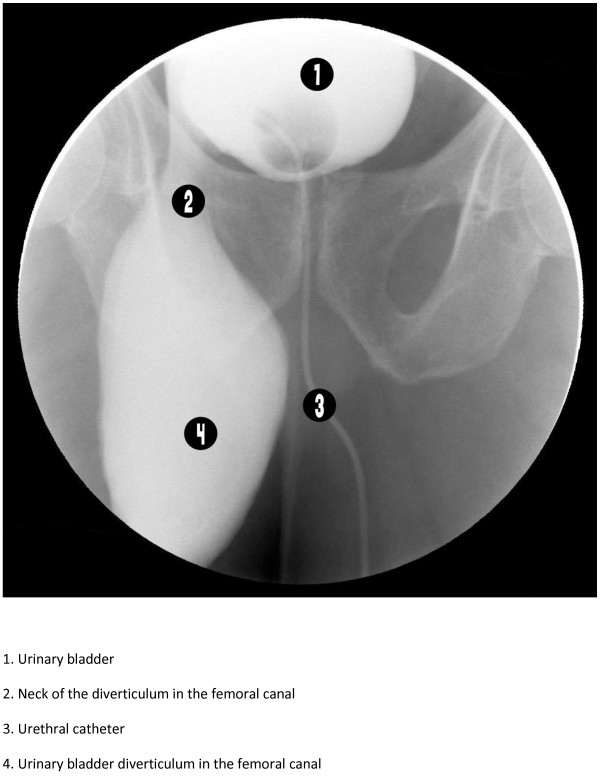
Retrograde urethrocystogram showing the urinary bladder diverticulum herniated in to the femoral canal.

**Figure 2:**
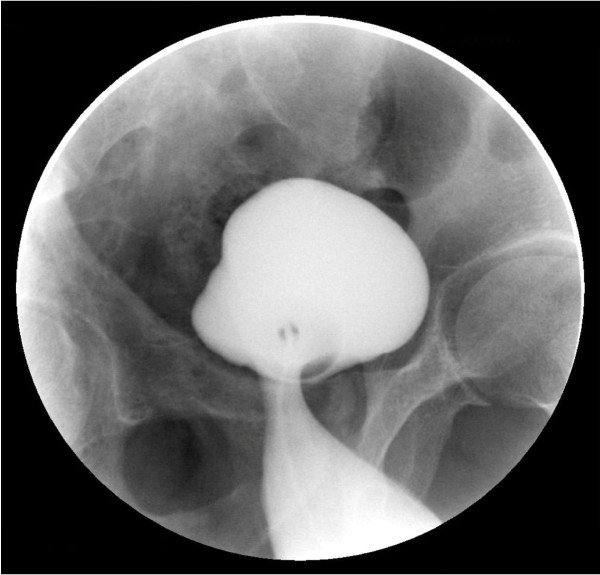
Oblique view of the urinary bladder and the diverticulum.

Abdominal ultrasound showed 11 × 5 cm bladder diverticulum herniated into the right groin region. The size of the prostate was estimated to be 60 grams and the post residual urine volume about 150 ml. Pelvic CT scan was requested but the patient refused to do it because of its cost. Cystogram was done to confirm the diagnosis and showed a bladder diverticulum herniated into the right femoral canal (Figures 1 and 2).

On planning for an emergency surgery, urine analysis, CBC, serum creatinine and urea, serum electrolytes, chest x-ray and ECG were all done and were within normal limits. The patient gave an informed consent only for diverticulectomy and hernia repair and preferred to try medical treatment for the benign prostatic hypertrophy.

Pfannenstiel incision was done, retroperitoneal space was opened, and dissection around the right side of the bladder revealed a congested urinary bladder diverticulum entrapped through the femoral ring which was dissected and reduced back with difficulty. Diverticulectomy was then performed and the femoral hernia was repaired using a polypropylene rolled plug mesh placement. During closure of the wound, a bulge was noticed in the right inguinal area. By palpation, it was proved to be reducible right inguinal hernia. Extension of the pfannenstiel incision to the right side, inguinal canal approached anteriorly opened, indirect inguinal hernia was found, hernia sac was dissected and excised. Hernia was repaired using a tensio and on free mesh technique. Prophylactic antibiotic (ceftriaxone) was given for 3 days. Foley’s catheter removed after 4 days and the patient was discharged.

Six months after surgery, none of the hernias recurred, but his lower urinary symptoms were only partially relieved by the medical treatment.

## Discussion

Hernias are usually the result of musculo-apponeurotic weakness or secondary to an increased intra-abdominal pressure. Patients with prostatic hypertrophy usually have increased intrarvesical pressure and at increased risk of the development of a bladder diverticula [4]. Femoral hernias are more often found in females and usually contain small intestine and omentum in their sacs. Reported uncommon contents include cecum, appendix, meckel’s diverticulum (Littre Hernia), testis, ovary, transverse colon and even stomach or kidney [5]. Urinary bladder diverticula can be contained in inguino-scrotal hernias. To the best of our knowledge, there has been only one case reported in the literature of a femoral hernia containing a urinary bladder diverticulum [6], (Table 1).

**Table 1:** Reported case of a right femoral hernia containing a urinary bladder diverticulum

**Number**	**Age**	**Sex**	**Side**	**Chronic dysuria**	**Author**	**Journal**	**Year**
1.	72	Male	Right	Present	N.P. Buchholz et al.	British Journal of Urology	1998
Present case	59	Male	Right	Present	Omari AK, Alghazo MA		

Bladder diverticula are usually caused by an increased intravesical pressure as a result of infravesical obstruction resulting from benign prostatic hypertrophy, urethral stricture, bladder neck contracture and others. In our case, the infravesical obstruction was caused by benign prostatic hypertrophy. A long standing history of difficulty of urination, incomplete voiding and straining in the setting of a groin hernia as seen in our case should increase the suspicion for the diagnosis of a sliding inguino-scrotal hernia containing the urinary bladder or a bladder diverticulum. The diagnosis of groin hernia is usually based on the clinical findings. However, it is important to know its exact location, its relationships, and the characteristics of its contents before planning surgical intervention [1].

As a noninvasive technique, several authors report the useful diagnostic application of ultrasonography in determining the contents of groin hernia [7,8].

In this case, ultrasonography showed the bladder diverticulum as a content of the groin hernia but did not provide solid information about its relationships. Nowadays, CT scan imaging is believed to be the study of choice in correctly localizing the groin hernia, in demonstrating its relationship with the inferior epigastric vessels and in the characterization of its contents [9,10]. We requested a CT scan study but the patient could not do it due to financial reasons. The diagnostic imaging used in the past, and even now, were intravenous urography and retrograde cystography and it was reported that a cystogram should be performed during the preoperative evaluation to assess the urinary bladder anatomy and the degree of its involvement [11,12]. After the nonconclusive findings of the ultrasound examination about the content and the exact relations of the hernia, we performed urgent retrograde cystogram which showed a huge urinary bladder diverticulum herniating into the femoral canal, a finding which was confirmed intra operatively. The urinary bladder diverticulum herniated into the femoral canal was associated with a reducible indirect inguinal hernia. Up to our knowledge, this combination had never been reported in the literature review.

The treatment of symptomatic bladder diverticula secondary to benign prostatic hypertrophy, either as a content of a hernia or not, is diverticulectomy and simple prostatectomy [13]. The surgical treatment of a bladder diverticulum herniated through the femoral or inguinal canals can be performed either by extra or intra peritoneal approaches. Regarding this case, we approached the femoral hernia posteriorly and extraperitonealy while the coexisted inguinal hernia was approached anteriorly through an extended Pfannenstiel incision. Prostatectomy was not performed respecting the patient wishes as he preferred medical treatment with alpha-blockers and 5-alpha reductase inhibitors.

## Conclusion

Urinary bladder diverticula should be considered as a possible content of femoral hernias especially in males with long standing obstructive lower urinary tract symptoms. As the clinical features of such a case are not specific, a high index of suspicion along with proper imaging studies are of great help in making a timely diagnosis to improve the outcome. Combined femoral hernia containing a bladder diverticulum with an inguinal hernia is a possible entity.

### Consent

Written informed consent was obtained from the patient for publication of this case report and accompanied images. A copy of the written consent is available for review by the Editor-in-Chief of this journal.

### Ethical approval

Institution Review Board (IRB) of the Jordan University of Science and Technology and King Abdallah University Hospital granted the approval for all the work done in these institutions.

## Competing interests

The authors declare that they have no competing interests.

## Authors’ contributions

AO: participated in the design and coordination of the study and helped to draft the manuscript and reviewed the literature. MA: participated in the design, studied the images and reviewed the literature. Both authors read and approved the final manuscript.
